# Long-term outcome after repair of interrupted aortic arch in a single centre^†^

**DOI:** 10.1093/icvts/ivaf026

**Published:** 2025-02-12

**Authors:** Michaela Kreuzer, Eva Sames-Dolzer, Andreas Tulzer, Melanie Klapper, Roland Mair, Gregor Gierlinger, Fabian Seeber, Rudolf Mair

**Affiliations:** Division of Pediatric and Congenital Heart Surgery, Kepler University Hospital, Linz, Austria; Medical Faculty, Johannes Kepler University Linz, Linz, Austria; Division of Pediatric and Congenital Heart Surgery, Kepler University Hospital, Linz, Austria; Medical Faculty, Johannes Kepler University Linz, Linz, Austria; Medical Faculty, Johannes Kepler University Linz, Linz, Austria; Department of Pediatric Cardiology, Kepler University Hospital, Linz, Austria; Division of Pediatric and Congenital Heart Surgery, Kepler University Hospital, Linz, Austria; Division of Pediatric and Congenital Heart Surgery, Kepler University Hospital, Linz, Austria; Medical Faculty, Johannes Kepler University Linz, Linz, Austria; Division of Pediatric and Congenital Heart Surgery, Kepler University Hospital, Linz, Austria; Medical Faculty, Johannes Kepler University Linz, Linz, Austria; Division of Pediatric and Congenital Heart Surgery, Kepler University Hospital, Linz, Austria; Medical Faculty, Johannes Kepler University Linz, Linz, Austria; Division of Pediatric and Congenital Heart Surgery, Kepler University Hospital, Linz, Austria; Medical Faculty, Johannes Kepler University Linz, Linz, Austria

**Keywords:** interrupted aortic arch, aortic arch reconstruction

## Abstract

**OBJECTIVES:**

Mortality in patients with interrupted aortic arch (IAA) rates among the highest in congenital heart surgery. Various techniques for aortic arch repair are described; reintervention rates remain substantial. This retrospective single-centre study aimed to evaluate the long-term outcome in a biventricular series with a preferably performed direct anastomosis and one-stage repair.

**METHODS:**

At the Children’s Heart Center Linz, 58 biventricular patients with IAA were operated between 1999 and 2023. Median age at operation was 10 [7; 15] days and weight was 3.3 [3; 3.7] kg. In the 24 children with ventricular septal defects (VSDs) only, the arch was repaired by a direct anastomosis. Thirty-four had complex concomitant heart defects, and the arch reconstruction was performed by direct anastomosis (20), direct anastomosis + patch (10), reverse subclavian flap + patch (3) and aortic autograft + patch (1).

**RESULTS:**

Median cardiopulmonary bypass time was 222 [159; 315] min, and aortic cross-clamp time was 94 [75; 143] min. Two patients died during the hospital stay (4%), two patients after discharge (4%), and four (7%) required an arch reintervention during a follow-up period of median 9.3 [6.2; 17.2] years. There was no death or arch reintervention in the VSD group.

**CONCLUSIONS:**

All-cause mortality in biventricular patients with IAA was 7%, and the arch reintervention rate was 8%. Direct aortic anastomosis in patients with VSD can only be performed with excellent outcomes, with no deaths or arch reinterventions being observed after a follow-up up to 24 years.

**Clinical registration number:**

EK Nr 1268/2021, Ethics committee of the Medical Faculty at Johannes Kepler University Linz.

## GRAPHICAL ABSTRACT

**Figure:**
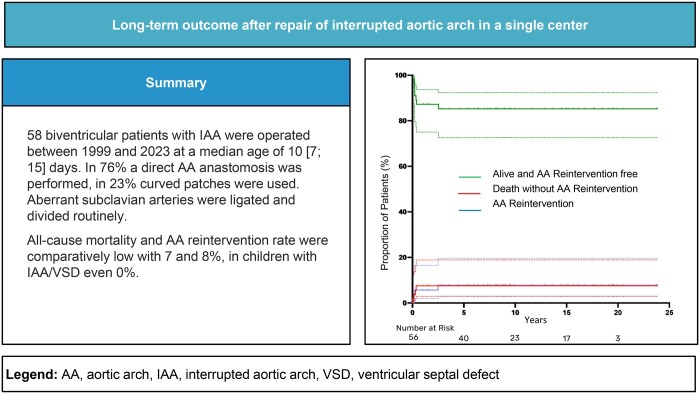

## INTRODUCTION

The interrupted aortic arch (IAA) is a rare congenital heart defect and associated with either only a ventricular septal defect (VSD) or with complex cardiac anomalies. There are several publications of single-centre series describing various surgical strategies [1–3]. Early and late mortality as well as reintervention rates on the aortic arch (AA) remain high [4, 5]. The aim of this retrospective single-centre study was to evaluate the mortality and reintervention rate in a biventricular series with a preferably performed direct anastomosis and one-stage repair.

## PATIENTS AND METHODS

### Patients

Between 1999 and 2023, a total of 65 patients with IAA were operated at the Children’s Heart Center Linz; of these, 58 patients had a biventricular circulation. This is our study cohort. The patients’ characteristics are provided in Table 1. There was no case with a right AA. According to the underlying disease, patients were divided into two anatomical subgroups: 24 children had an IAA and a VSD only (‘IAA/VSD’ group); the remaining 34 children were summarized as the ‘complex’ group. All patients were operated by a complete repair as newborns without any previous intervention except four: two girls out of the complex group came from abroad with previous pulmonary artery banding or pulmonary artery banding and ductal stenting. They were operated by a complete repair at the age of 54 and 55 days. Another girl from abroad with IAA/VSD showed up at the age of 17 months without any previous intervention. Diagnostic catheterization showed reversible pulmonary hypertension, and she was operated by a complete repair at the age of 546 days. Only one preterm baby with a birth weight of 2.4 kg was provided with a pulmonary artery banding and ductal stenting at our centre. The VSD closure and AA repair were then performed at the age of 128 days. The IAA/VSD group was significantly older than the complex group. This fact is not due to a specific decision of our centre, but to the later referral of these patients.

**Table 1:** Patients’ characteristics

Subgroup	IAA/VSD	Complex group	Total	*P* value
Patients (female)	24 (4)	34 (16)	58 (20)	
Age (days), range	12 [9; 19], 2–546	7 [7; 12], 3–55	10 [7; 15], 2–546	0.025
Weight (kg), range	3.4 [3.1; 3.8], 2.3–8.6	3.3 [3; 3.7], 2.3–5.1	3.3 [3; 3.7], 2.3–8.6	0.467
Additional cardiac malformation	– VSD (24)	– VSD/AS (11) – VSD/subvalvular AS (3) – Truncus arteriosus (6) – Taussig Bing (2) – TGA or ccTGA/VSD (3) – AP window (4) – Complete AVC (2) – DORV/AP window/ARCAPA (1) – VSD/PS/atrial septal aneurysm (1) – AS/unbalanced partial AVC/unroofed coronary sinus/interrupted inferior vena cava (1)	– VSD (24) – VSD/AS (11) – VSD/subvalvular AS (3) – Truncus arteriosus (6) – Taussig Bing (2) – TGA or ccTGA/VSD (3) – AP window (4) – Complete AVC (2) – DORV/AP window/ARCAPA (1) – VSD/PS/atrial septal aneurysm (1) – AS/unbalanced partial AVC/unroofed coronary sinus/interrupted inferior vena cava (1)	n.e.
Additional vascular anomalies	– Aberrant right subclavian artery (1)	– Aberrant right subclavian artery (8) – ARCAPA (2) – Right subclavian artery out of right PA (1) – Left subclavian artery out of left PA (1) – Right PA out of ascending aorta (1)	– Aberrant right subclavian artery (9) – ARCAPA (2) – Right subclavian artery out of right PA (1) – Left subclavian artery out of left PA (1) – Right PA out of ascending aorta (1)	n.e.
Interrupted AA type	A (5)	A (12)	A (17)	0.260
	B (19)	B (22)	B (41)	
	C (0)	C (0)	C (0)	
Genetic disorders	13 (54%)	13 (39%)	26 (45%)	0.288
− Di George syndrome	11 (46%)	7 (21%)	18 (31%)	
− Down syndrome	0 (0%)	2 (6%)	2 (3%)	
− Kabuki syndrome	1 (4%)	0 (0%)	1 (2%)	
− Hanhart syndrome	0 (0%)	1 (3%)	1 (2%)	
− 4q33 Deletion	0 (0%)	1 (3%)	1 (2%)	
− Heterotaxy syndrome	0 (0%)	1 (3%)	1 (2%)	
− Multiple malformation syndrome (no genetic examination)	1 (4%)	1 (3%)	2 (3%)	
Previous operation	– PAB+ductal stent (1)	– PAB+ductal stent (1)	– PAB+ductal stent (2)	>0.999
		– PAB (1)	– PAB (1)	

Data are presented as the median (first, third quartile) or numbers. AA: aortic arch; AP: aortopulmonary; ARCAPA: anomalous right coronary artery origin from pulmonary artery; AS: aortic stenosis; AVC: atrioventricular canal; ccTGA: congenitally corrected transposition of the great arteries; DORV: double-outlet right ventricle; n.e.: not evaluated; PA: pulmonary artery; TGA: transposition of the great arteries. *P* value: VSD/IAA versus complex group.

### Operative technique

In the preoperative planning, a transthoracic echocardiography was carried out routinely. An additional angiography was performed in the case of residual uncertainties. The approach for all 58 surgeries was a median sternotomy. We started the cardiopulmonary bypass by double arterial and venous cannulation in all cases: the first arterial canula was put into the innominate or right carotid artery, the second into the pulmonary bifurcation. The AA repair of the first patient of our cohort was performed during hypothermic circulatory arrest. Then, antegrade cerebral perfusion was established at our centre, and we used this method for the next eight patients with IAA. In October 2003, we started to perform all AA reconstructions during whole-body perfusion by using the descending aorta for the second arterial canula [6]. This was the case in 84% (*n* = 49) of this study cohort. Perioperative details and concurrently performed procedures are presented in Table 2.

**Table 2:** Peri- and postoperative data

Subgroup	IAA/VSD	Complex group	Total	*P* value
Patients	24	34	58	
Aortic arch repair				
− Direct anastomosis	− 24 (100%)	− 20 (59%)	− 44 (76%)	<0.001
− Curved patch		− 10 (29%)	− 10 (17%)	
− +Rev subclavian flap		− 3 (9%)	− 3 (5%)	
− +Aortic autograft		− 1 (3%)	− 1 (2%)	
Ligation and division of				n.e.
− Aberrant right subclavian artery	− 1 (4%)	− 9 (26%)	− 10 (17%)	
− Left subclavian artery	− 12 (50%)	− 13 (38%)	− 25 (43%)	
Concurrently performed procedures	VSD closure (24)	– VSD closure (25) – Ross-Konno (12) – TAC repair (6) – AP window closure (4) – Modified Konno (3) – Arterial switch (3) – Complete AVC repair (2) – Pulmonary artery banding (ccTGA, 2) – Pulmonary valve or artery reconstruction (2) – Truncal valve repair/replacement (2) – Systemic AV valve repair (1) – Norwood/Rastelli (1) – Coronary artery transfer (1)	– VSD closure (49) – Ross-Konno (12) – TAC repair (6) – AP window closure (4) – Modified Konno (3) – Arterial switch (3) – Complete AVC repair (2) – Pulmonary artery banding (ccTGA, 2) – Pulmonary valve or artery reconstruction (2) – Truncal valve repair/replacement (2) – Systemic AV valve repair (1) – Norwood/Rastelli (1) – Coronary artery transfer (1)	n.e.
Bypass time (min)	168 [154; 192], 22 pts	301 [236; 332], 34 pts	222 [159; 315], 56 pts	<0.001
Aortic cross-clamp time (min)	74 [68; 88]	139 [100; 163]	94 [75; 143]	<0.001
Hypothermic circulatory arrest (min, 1 pt)	27	–	–	n.e.
Delayed sternal closure	3 (13%)	16 (47%)	19 (33%)	n.e.
ECMO postoperatively	0 (0%)	4 (12%)	4 (7%)	0.134
Stay on ICU (d)	9 [8; 15], 22 pts	17 [9; 25], 30 pts	13 [8; 23], 52 pts	0.078
In-hospital stay (d)	25 [16; 43], 22 pts	26 [20; 33], 28 pts	26 [19; 37], 50 pts	0.957

Values are presented as medians and quartiles 1 and 3 or numbers. AA: aortic arch; AP: aortopulmonary; AV: atrioventricular; AVC: atrioventricular canal; d: days; IAA: interrupted aortic arch; min: minute; n.e.: not evaluated; pts: patients; rev: reverse. *P* value: IAA/VSD versus complex group.

**Figure 1:**
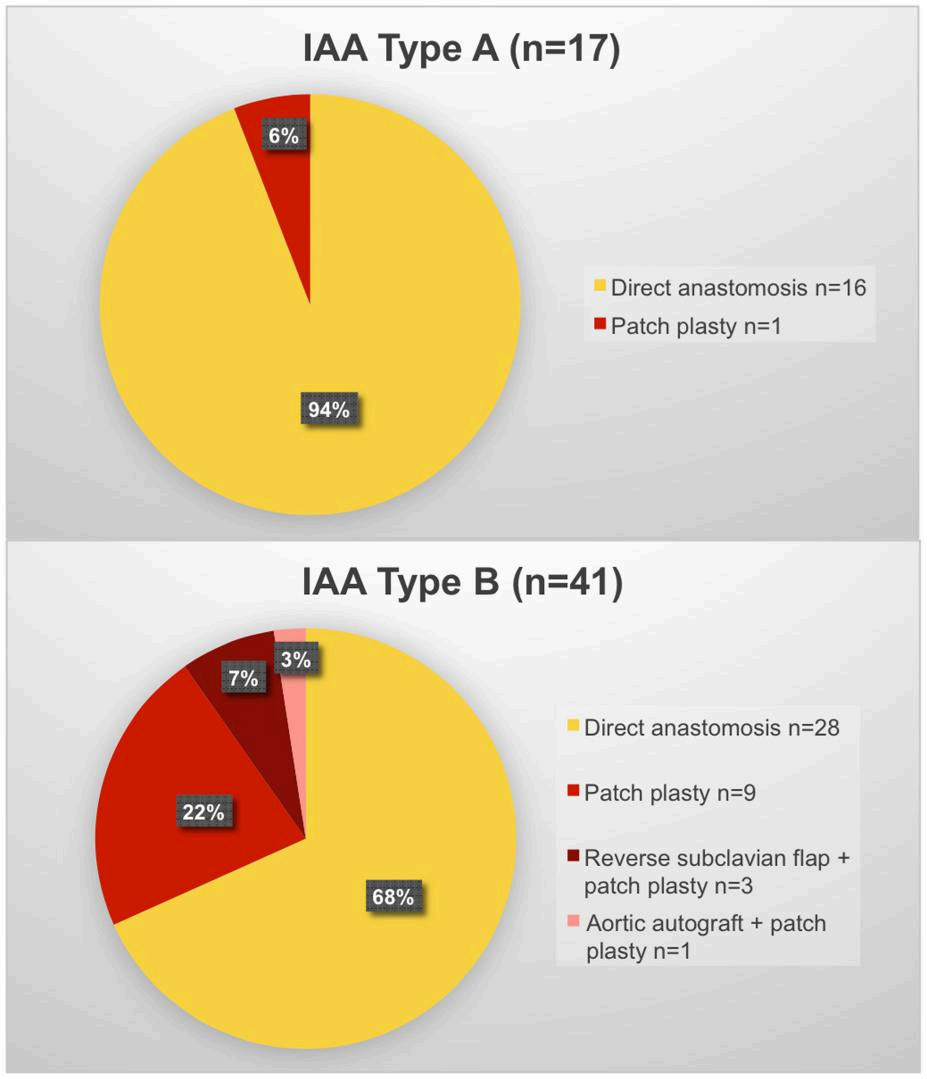
Surgical strategy of AA repair according to IAA type

The preferred method for AA reconstruction was a direct anastomosis without any patch material, which was the case in all IAA/VSD patients. In total, it was performed in 44 children (76%, Fig. 1). Exceptions were 83% of patients with concurrently performed Ross-Konno procedures (*n* = 10), 50% of truncus arteriosus communis patients (*n* = 3) and one complex case of an aortopulmonary window. For the patch plasty, only patches curved in two planes were used, either out of an aortic or pulmonary homograft, a polytetrafluorethylene vascular prosthesis [7] or the patient’s pericardium. The latter was formed by binding the pericardium tightly around a metal ring. Then it was fixated by glutardialdehyde (0.65%) for 20 min followed by three times of 2 min washing in Ringer’s solution (Supplementary Fig. S1). An additional autograft out of the ascending aorta for the convexity of the distal arch or a reverse subclavian flap was used in three Ross-Konno patients and one truncus arteriosus communis patient.

Aberrant right subclavian arteries were present only in cases with IAA type B (*n* = 10, 24%). They were ligated and divided routinely. In 28 patients with an IAA type B (68%), the left subclavian artery was intraoperatively assessed as interfering with a tension-free arch repair or used as a reverse subclavian flap and therefore ligated and divided. In three patients, it was reconnected to the convexity of the AA or to the left carotid artery.

### Statistics

This retrospective study was approved by the ethics committee of the Medical Faculty at Johannes Kepler University Linz on 27 June 2024 (EK Nr: 1268/2021). Informed consent was waived because of the retrospective nature of the study and the use of pseudonymized clinical data for the analysis.

All data of continuous variables were checked for normal distribution (test of normality: Kolmogorov–Smirnov with Lilliefors significance correction, type I error = 10%). The Cox-continuous variables with normally distributed data were compared between subgroups by the *t*-test for independent samples. For comparisons of continuous variables without normally distributed data, the exact Mann–Whitney *U*-test was used. Dichotomous variables were compared by the Fisher’s exact test, other categorical variables (nominal variables with >2 categories) by the exact chi-square test (with provision of adjusted residuals). For the comparison of the occurrence of events, depicted by Kaplan–Meier plots, the log-rank test was used. The type I error was not adjusted for multiple testing. Therefore, the results of inferential statistics are descriptive only. Statistical analyses were performed using the open-source R statistical software package, version 4.3.1 (The R Foundation for Statistical Computing, Vienna, Austria). In the case of missing values, the number of patients with available data was added to the tables or within the text.

## RESULTS

### Postoperative data and early mortality

Postoperative data are presented in Table 2. Fifty-six patients (97%) could be discharged from hospital; two patients (3%) died during the stay on intensive care unit (ICU). Both were out of the complex group. Patient 18 was a girl with DiGeorge syndrome, double-outlet right ventricle, aortic atresia, non-committed VSD, aortopulmonary window, anomalous right coronary artery origin from pulmonary artery, IAA type B and an aberrant right subclavian artery. She was provided with a Norwood/Rastelli operation, ligation of the right subclavian artery and a direct AA anastomosis. She had a prolonged stay on ICU with twice a laparotomy because of bleeding and twice a cardiac reintervention because of peripheral pulmonary and left bronchial stenosis, which was diagnosed by magnetic resonance imaging. At first, the right ventricle to pulmonary artery (RVPA) conduit was shortened, and during the second reoperation, a Lecompte maneuvre was performed. Unfortunately, the girl died on postoperative day (POD) 117 due to complications of long-term ventilation and with a brain atrophy. Patient 57 was a girl with a heterotaxy syndrome, aortic stenosis, left ventricular outflow tract obstruction, IAA type B, unbalanced partial atrioventricular canal, unroofed coronary sinus, left atrial isomerism, left superior vena cava and an interrupted inferior vena cava with hemiazygos continuation. At the age of 7 days, a Ross-Konno procedure and a sophisticated AA repair by using an aortic autograft, a patch plasty and ligation of the left subclavian artery were performed. The intraoperative course was complicated with twice aortic cross clamping for AA repair and a second time cardiopulmonary bypass for revision of the RVPA conduit. She needed extracorporeal membrane oxygenation (ECMO) postoperatively and died due to multiorgan failure on POD 5.

### Complications

In 4 out of 58 children (7%), a postoperative laryngeal nerve paralysis was found, and in 2 of them, it was only temporary. The two patients with a persistent paralysis did not show major clinical symptoms at their last follow-up. There were two cases of bronchomalacia with prolonged ventilation time, and one patient was provided with a tracheostoma because of a subglottic membrane and hypoplastic vocal cords. There were two cases with epileptic seizures postoperatively. Two patients needed a pacemaker implantation, one a ligation of the thoracic duct because of prolonged effusion. There was one case of short postoperative peritoneal dialysis due to temporary renal failure.

### Long-term outcome and late mortality

Long-term outcome was available in 54 patients; 2 children from abroad were lost to follow-up. An overview of the most important results is provided in Table 3. After a median follow-up period of 9.3 [6.3; 17] years, 52 patients (93%) were alive, and 2 children died after hospital discharge. One girl with truncus arteriosus communis deceased unexpectedly at home on POD 54. Another boy with IAA/VSD/pulmonary stenosis/atrial septal aneurysm, Hanhardt syndrome and multiple additional malformations (hypoglossia, hypodactyly, peromelia, micrognathia, etc.) experienced several extracardiac hospital admissions and passed away on POD 139 most likely due to pneumonia.

**Table 3:** Results

Subgroup	IAA/VSD	Complex group	Total	*P* value
In-hospital mortality	0 (0%)	2 (6%)	2 (4%)	0.504
Late mortality	0 (0%)	2 (6%)	2 (4%)	0.501
All-cause mortality	0 (0%)	4 (12%)	4 (7%)	0.127
Follow-up period (years)	12.6 [5.9; 19.1] 24 patients	9 [6.2; 14.7] 28 patients	9.3 [6.2; 17.2] 52 patients	0.202
Transcatheter reintervention on AA	0 (0%)	3 (11%)	3 (6%)	n.e.
Surgical reintervention on AA	0 (0%)	2 (7%)	2 (4%)	n.e.
Any reintervention on AA	0 (0%)	4 patients (14%)	4 patients (8%)	0.127

Values are presented as medians and quartiles 1 and 3 or numbers. AA: aortic arch; n.e.: not evaluated. *P* value: IAA/VSD versus complex group.

**Figure 2:**
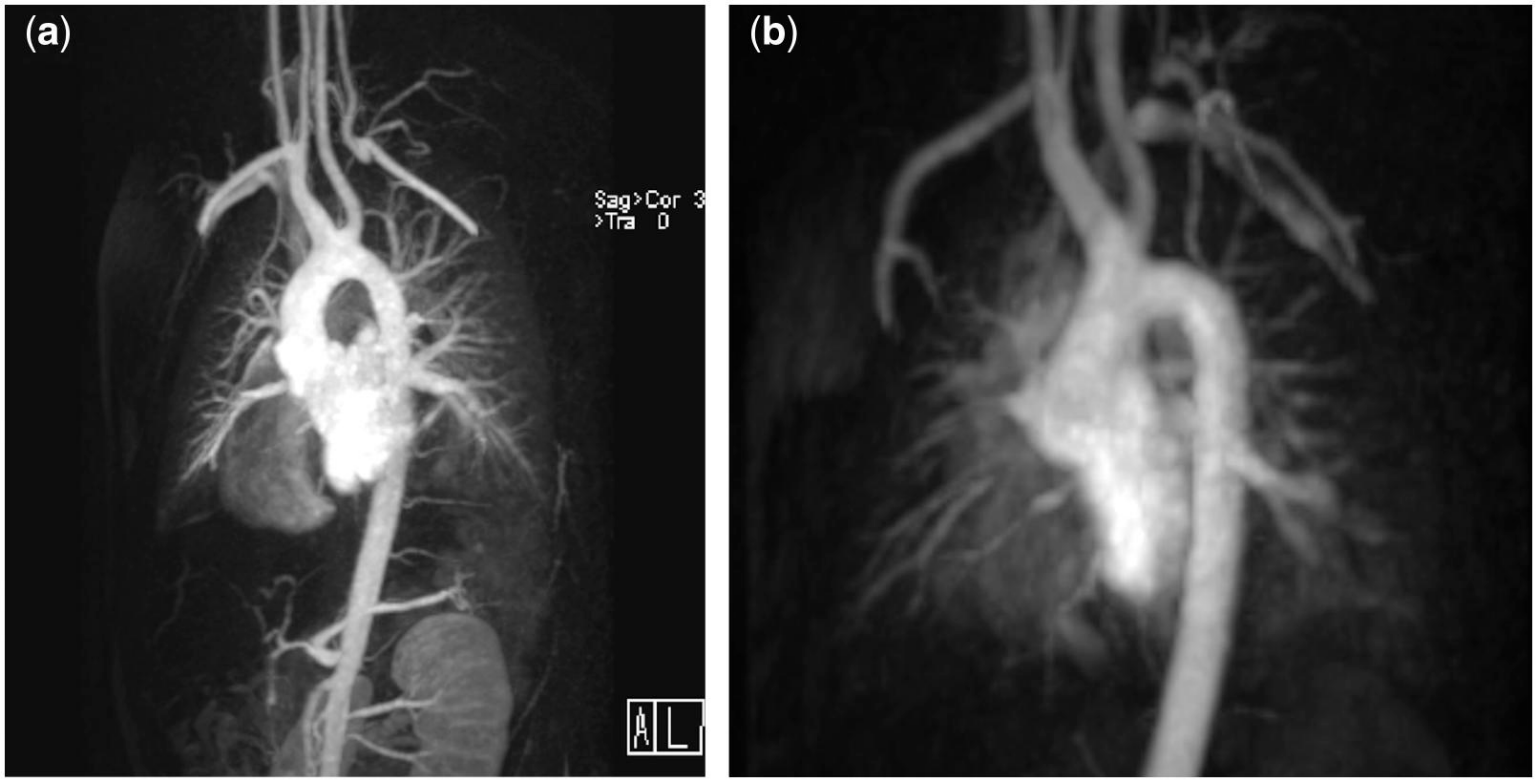
(a) Magnetic resonance imaging 15 years after direct AA anastomosis, ligation and division of the left subclavian artery and VSD closure in a patient with IAA type B, VSD and bicuspid aortic valve. (b) 11 years after Rastelli procedure, AA patch plasty (curved polytetrafluorethylene patch), ligation and division of the left subclavian artery in a patient with truncus arteriosus communis type A1 (Van Praagh) and IAA type B

We aimed for round and short arches; images are provided as Fig. 2a and b. Two patients (4%), both out of the complex group, needed a surgical reintervention on the AA; in one of them, a transcatheter balloon angioplasty had been tried earlier. The first recurrent obstruction was repaired by a patch plasty 73 days and the second by an extended end-to-end anastomosis 3 years after the first surgery. Two further complex patients with recurrent arch stenosis were treated by transcatheter intervention, one by dilation 63 days and one by a stent implantation 68 days postoperatively. In 47/52 patients, no gradient at the AA was seen at the last follow-up echocardiography. In five patients, flow accelerations of 2.4–3.1 m/s, but normal flow patterns in the abdominal aorta were assessed. The case of 3.1 m/s is the above-mentioned patient with an isthmus stent and will surely need a dilation of the stent in the near future.

**Figure 3:**
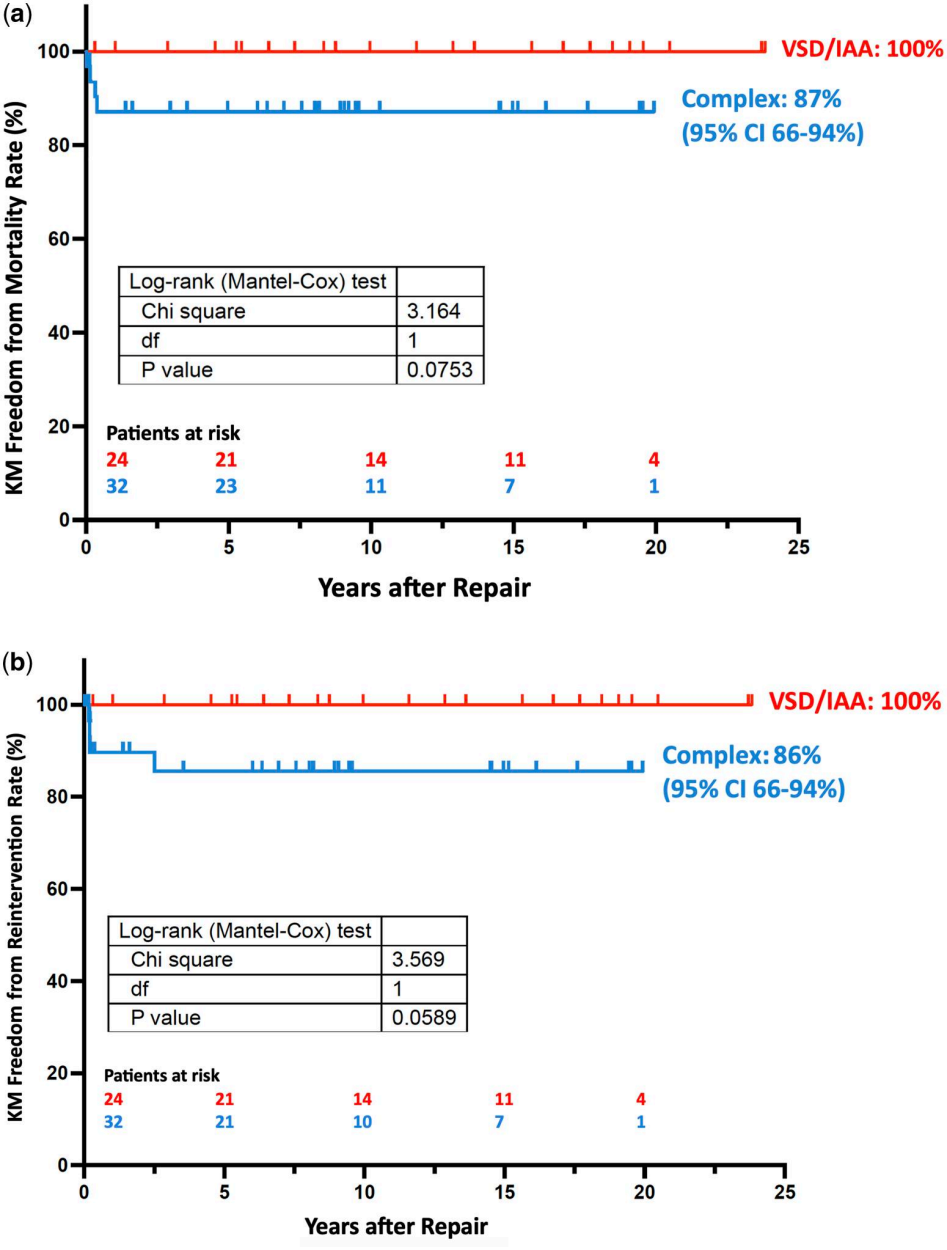
(a) Kaplan–Meier curve regarding survival in the IAA/VSD versus complex group. (b) Kaplan–Meier curve regarding time to surgical or transcatheter aortic arch reintervention in the IAA/VSD versus complex group

The Kaplan–Meier curve regarding survival is provided as Fig. 3a, regarding time to any reintervention on the AA as Fig. 3b. The influence of the variables ‘subgroup’ (IAA/VSD versus complex), ‘date of operation’, ‘age at operation’, ‘aberrant subclavian artery’, ‘patch plasty’, ‘polytetrafluorethylene used for patch plasty’, ‘IAA type’ (A versus B) and ‘any syndrome’ on the occurrence of ‘exitus’, ‘any AA reintervention’ and ‘exitus or AA reintervention’ was investigated by Cox regression analyses (proportional hazards assumption assessed via the Schoenfeld residuals). Only IAA type A showed a positive impact on ‘time to any AA reintervention’ (*P* = 0.023). VSD/IAA versus the complex group only showed a significant statistical difference in ‘exitus or AA reintervention’ (*P* = 0.008).

There were several planned or unplanned surgical reinterventions on other cardiac structures: RVPA conduit exchange (6), left (1) or right (3) pulmonary artery patch plasty, subaortic stenosis repair (4), patch plasty of ascending aorta (4), truncal valve replacement (2), aortic valve repair (1), Ross procedure (1), David’s procedure (1), mitral valve repair (2), residual VSD closure (1) and double switch operation (2). Transcatheter reinterventions addressed stenosis of the left (3) or right (3) pulmonary artery, RVPA conduit (6) and aortic valve (1). Eleven patients needed more than one reintervention; in most cases, combined procedures were performed. In total, 30 out of 52 patients with available follow-up data (58%) did not have any cardiac reintervention so far. This was the case in 13/28 (46%) out of the complex group and 17/24 (71%) out of the IAA/VSD group.

There was one case of left bronchial stenosis in this study. The very complex case is described in detail in the ‘Postoperative data and early mortality’ section (patient 18).

## DISCUSSION

There are many publications presenting single-centre results of cases with IAA [1–3, 8]. Series including univentricular patients have shown worse results than biventricular series, especially regarding mortality [2, 3]. This is an expected finding due to the completely different physiology of these patients with many additional problems besides the IAA. Therefore, the univentricular cases were excluded from the current study.

**Figure 4:**
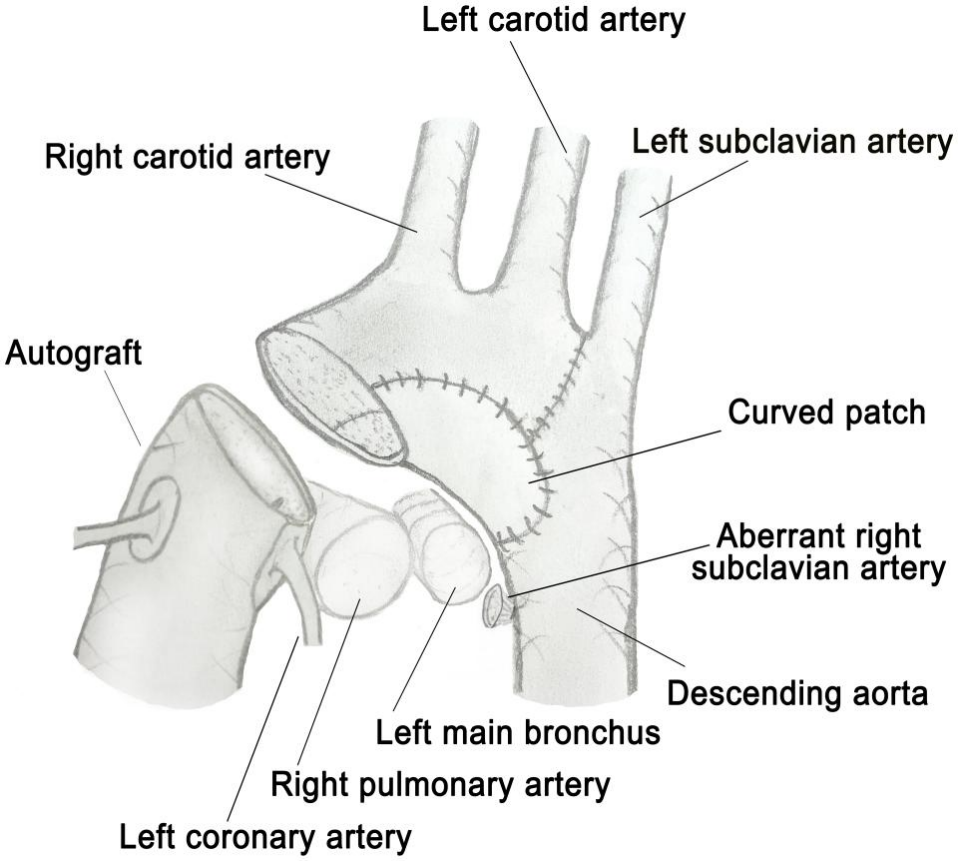
Technique of aortic arch patch plasty in Ross-Konno procedures with interrupted aortic arch type B. The aberrant right subclavian artery was ligated and divided

The single-centre study including the highest number of biventricular cases with IAA so far was published by Andrianova *et al.* recently [1]. They also compared the results of different series including their own and found early mortality rates of biventricular patients between 7.7% and 16.7% and late mortality between 2.8% and 19%. The early and late mortality of our cohort were 4% each, which is in summary superior to the mentioned results. Additionally, our reintervention rate on the AA of 8% is comparably low and the occurrence of a left bronchial stenosis rare. McCrindle *et al.* [4] found a higher AA reintervention rate when performing other than direct anastomosis with patch augmentation; other authors showed good results with using no patches for arch reconstruction [1, 8]. We share the opinion that it is important to use standardized techniques as far as possible. Our preferred method was a direct anastomosis using a resorbable 6/0 suture. A patch plasty was mainly performed in Ross-Konno patients and was done in analogy to a Norwood patch augmentation (Fig. 2b) [7, 9]. We prefer this technique due to the higher ostium of the left coronary artery after reimplantation as a U-shaped patch and in order to prevent circumferential redundancy due to the large diameter of the autograft in these cases (Fig. 4). If a direct anastomosis was made, the coronary artery would be located even more distally at the inner curvature of the AA. The left coronary and the pulmonary artery were then in great danger of being compressed between the aorta and the left main bronchus. A patch plasty was sparsely used in other complex cases with IAA type B (see ‘Operative technique’ section), if the distance was regarded as too large. Retrospectively, we checked the influence of the variables ‘subgroup’ (IAA/VSD versus complex), ‘Ross-Konno’ and ‘IAA type’ (A versus B) on the use of a patch plasty by logistic regression analysis. A positive effect was shown by the variable ‘Ross-Konno’ only (*P* < 0.001).

The Melbourne group found a high prevalence of early arch re-obstruction in patients with anomalous right subclavian artery and a negative impact of this vessel on long-term mortality within the group of children with VSD/IAA [1, 10]. Although they described different surgical strategies on dealing with an aberrant subclavian artery, they always preserved it. In our institution, it is ligated and divided routinely, and statistically, we did not see a negative impact of its presence on any outcome results. We also disconnected the left subclavian artery of 22 patients with IAA type B to enable a tension free anastomosis. Although the ligation of a vessel is never an easy decision, the findings of the Melbourne group together with our results seem to confirm our strategy. In three cases, the left subclavian artery was reconnected to the aorta or carotid artery. However, a routine follow-up imaging of one of these three patients showed that the vessel was closed at the anastomosis site. Several studies describe reduced arm length, circumference, arm muscle mass or grip strength after ligation of a subclavian artery [11–13]. However, no significant functional differences in adulthood were found between groups after subclavian flap repair versus end-to-end aortic anastomosis during childhood [13]. At the last follow-up examination of our patients after ligation of the subclavian artery, no exercise anomalies or any impact on quality of life were mentioned.

Over the past decades, a one-stage repair has become standard at many departments [1–3], and the same is the case at our centre [14]. We are of the opinion, that the chance for complications increases with longer duration of a palliated circulation. Furthermore, we regard an AA repair after ductal stenting as very challenging, and we often see problems with the pulmonary arteries after bilateral banding. In this series, the only exception from a single-stage repair was one preterm girl (see ‘Patients’ section). She needed a stent due to a left pulmonary artery stenosis later on. Further limitations would be low-weight babies (<2000 g) and serious cerebral, renal or abdominal complications preoperatively.

The limitations of this study are its retrospective nature, the small number of patients and the lack of a control group. Further surveys with also longer follow-up periods will be necessary and useful.

## CONCLUSION

In the present study, patients with IAA could be treated with a low AA reintervention rate and mortality, also in complex biventricular patients. Children with VSD/IAA performed best without any death or need for arch reintervention during follow-up. A direct arch anastomosis was performed in 76% of the cohort; the residual patients were provided with curved patches. The routine ligation and division of each aberrant subclavian artery or an interfering left subclavian artery in IAA type B patients may have a positive impact on the low AA reintervention rate.

## Supplementary Material

ivaf026_Supplementary_Data

## Data Availability

The data underlying this article will be shared on reasonable request to the corresponding author.

## ABBREVIATIONS

AA: Aortic arch
ECMO: Extracorporeal membrane oxygenation
IAA: Interrupted aortic arch
ICU: Intensive care unit
POD: Postoperative day
RVPA: Right ventricle to pulmonary artery
VSD: Ventricular septal defect
