# Supplementary material for: Long-term outcome after repair of interrupted aortic arch in a single centre
Source: Interdiscip Cardiovasc Thorac Surg. 2025 Feb 12;40(2):ivaf026. doi: 10.1093/icvts/ivaf026 (PMC11879088; doi:10.1093/icvts/ivaf026)
Supplement: ivaf026_Supplementary_Data [file ivaf026_supplementary_data.zip › IAA FigureS1neu.pdf]

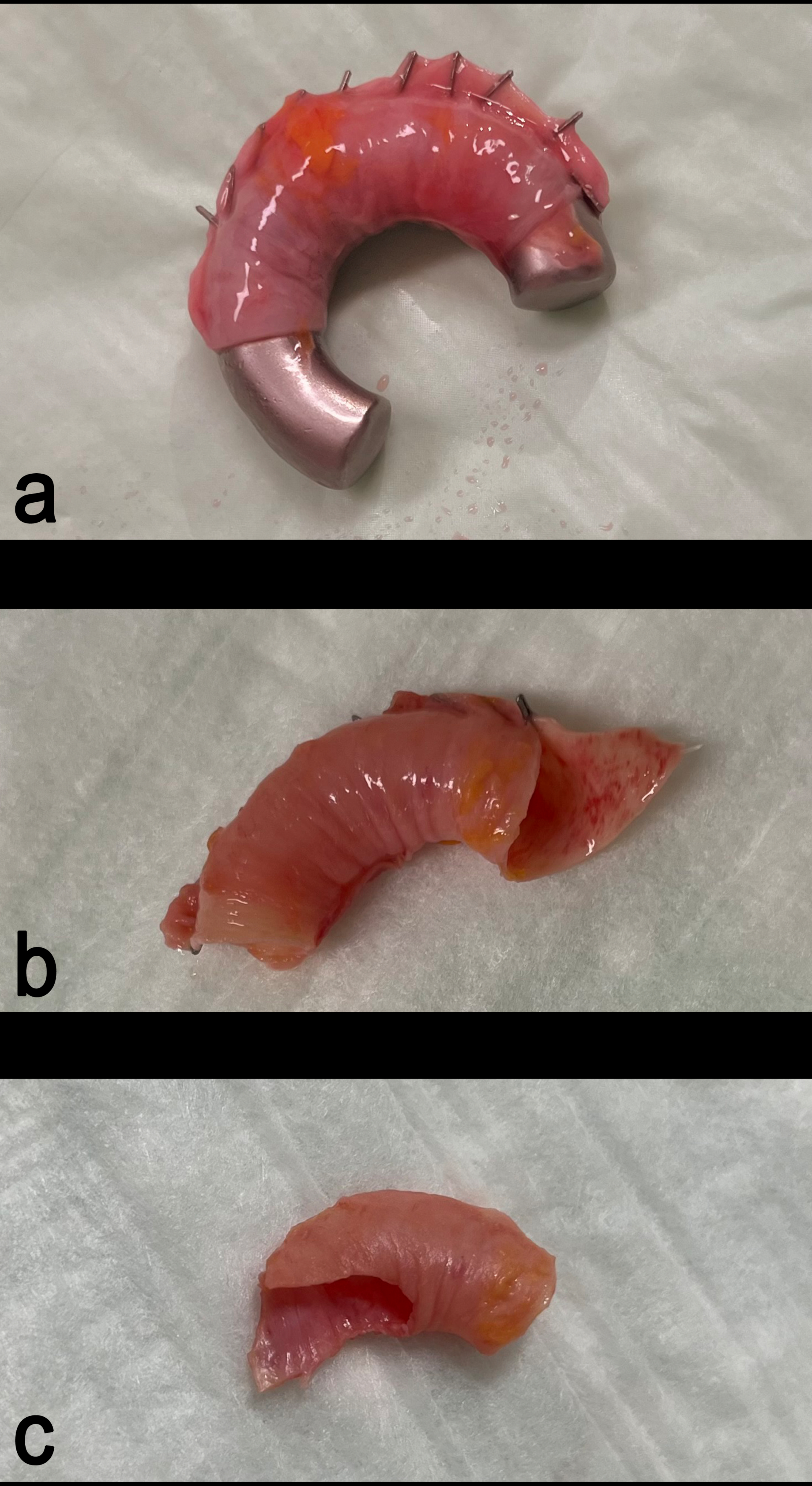

Supplementary figure S1: Preparation of a pericardium patch curved in two planes for aortic arch reconstruction.

a: Pericardium prepared for fixation by glutardialdehyd. Metal ring dimensions: inner diameter 30mm, thickness 8mm.

b: Fixated and washed patch after removing from metal ring.

c: Trimmed patch ready for implantation.
